# Availability, Promotion, and Signs of Alcohol Consumption: A Mixed Methods Study of Perceived Exposure and Objective Measures

**DOI:** 10.3390/ijerph17218153

**Published:** 2020-11-04

**Authors:** Andrea Pastor, Irene Molina de la Fuente, María Sandín Vázquez, Paloma Conde, Marina Bosque-Prous, Manuel Franco, Niamh Shortt, Xisca Sureda

**Affiliations:** 1 Public Health and Epidemiology Research Group, School of Medicine, University of Alcalá, Campus Universitario—Crta. de Madrid-Barcelona, 28871 Madrid, Spain; andrea.pastor@uah.es (A.P.); i.molina@edu.uah.es (I.M.d.l.F.); maria.sandin@uah.es (M.S.V.); p.conde@uah.es (P.C.); manuel.franco@uah.es (M.F.); 2 Malaria and NDTs Laboratory, National Centre of Tropical Medicine, Institute of Health Carlos III, 28029 Madrid, Spain; 3 Department of Epidemiology & Biostatistics, Graduate School of Public Health & Health Policy, City University of New York, New York, NY 10017, USA; 4 Faculty of Health Sciences, Universitat Oberta de Catalunya, Rambla del Poblenou, 156 08018 Barcelona, Spain; mbosquep@uoc.edu; 5 Department of Epidemiology, Johns Hopkins Bloomberg School of Public Health, Baltimore, MD 21205, USA; 6 Centre for Research on Environment, Society and Health, School of GeoSciences, University of Edinburgh, EH9 3JW Edinburgh, Scotland; niamh.shortt@ed.ac.uk; 7 Tobacco Control Research Group, Institut d’Investigació Biomèdica de Bellvitge-IDIBELL, l’Hospitalet de Llobregat SAvinguda de la Granvia de l’Hospitalet, 199, L’Hospitalet de Llobregat, 08908 Barcelona, Spain; 8 Consortium for Biomedical Research in Respiratory Diseases (CIBER en Enfermedades Respiratorias, CIBERES), 28029 Madrid, Spain

**Keywords:** alcohol, mixed methods, photovoice, alcohol availability, alcohol promotion, signs of alcohol consumption

## Abstract

This study describes the alcohol environment comparing residents’ perceptions and objective measures in two different income-level districts. Measures were gathered between 2017 and 2018 in two districts with different income levels in Madrid, Spain. We obtained perceived measures using Photovoice. We procured objective measures through social systematic observation. Data were integrated using triangulation. Perceived and objective measures of the alcohol environment were characterized and compared in terms of alcohol availability, alcohol promotion, and signs of alcohol consumption. The integration was classified as agreement, partial agreement, or dissonance. The results related to alcohol availability and signs of its consumption showed high agreement. Availability was high in both areas, which was recognized by residents. Residents of the high-income district (HID) discussed fewer signs of alcohol consumption, whilst those in the low-income district (LID) reported extensive signs of consumption. Such observations agreed with the objective measures. There were dissonances between the approaches for alcohol promotion. Although the alcohol promotion was higher in HID according to the objective measures, it was deeply discussed by LID residents. Both methodologies helped us deepen the understanding of the alcohol environment. These results may help design more effective interventions to prevent hazardous drinking.

## 1. Introduction

Alcohol consumption is one of the leading factors associated with disability and death worldwide [1,2]. 

Although alcohol consumption in Mediterranean countries has decreased in the last years [3], the binge drinking episodes have increased [4,5]. Research suggests that those who engage in binge drinking episodes have lower risk perceptions related to alcohol [6]. Such risk perceptions may be related to physical features of the urban environment, including availability, promotion, and signs of alcohol consumption [7].

The influence of the physical environment on the decision-making process is supported by Social Cognitive Theory (SCT) [8,9]. This theory addresses the role of the broader environmental context (i.e., high availability of alcohol) in the decision-making process, rather than individual determinants alone (i.e., gender, ethnicity, or income) [10,11]. One of the learning processes related with SCT is the “automatic cognitive process” [12]. Previous studies had defined this process as a mediator in the associations between the ubiquitous presence of alcohol (e.g., alcohol cues at promotion) and the early alcohol use [13,14,15]. Thus, physical features of the alcohol environment could influence the normalization of alcohol consumption and the self-capacity of refusing to drink alcohol [8,16]. 

Quantitative studies have found a positive relationship between alcohol availability and promotion and the normalization of its consumption [17,18]. A higher availability of alcohol outlets increases the opportunity to socialize around alcohol [19,20]. These results are supported by qualitative studies [19,20,21,22,23]. Moreover, both quantitative and qualitative studies have shown that the exposure to alcohol promotion at street view or signs of alcohol consumption on streets increase the expectancies around alcohol consumption and promote unhealthy behaviors [21,22,23,24,25,26].

Alcohol exposure has been mostly measured using surveys [27,28], focus groups [29,30], or social systematic observation [5,31,32]. However, other methodologies such as Participatory Action Research (PAR) might show results that are difficult to be revealed by other methodologies [33]. One such approach is Photovoice, which involves the participants in a critical discussion about a specific issue through the use of photography [34,35,36,37]. This methodology had been used previously among people with alcoholic problems [37]. Participants identified that high exposure to alcohol availability and promotion were hindering their recovery.

The combination of different approaches of alcohol environment through mixed methods may help deepen the understanding of the alcohol environment [38,39,40,41,42] and identify the needs in future alcohol control interventions [40,43].

In this study, we describe the alcohol environment in terms of alcohol availability, promotion, and signs of alcohol consumption through residents’ perceptions (using Photovoice) and objective measures (using social systematic observation) in two income diverse areas. Secondly, we compared similarities and differences between both approaches and across study areas.

## 2. Materials and Methods

### 2.1. Study Setting

This study was conducted in the city of Madrid, in two different income-level districts: “Chamberí” (high-income level district, HID) and “Villaverde” (low-income level district, LID). We based this study in these areas because of their high social participation detected in previous Photovoice projects [44,45].

We extracted the municipal data from the Madrid Municipal Registry (https://www.madrid.es/portales/) updated to January 2018. The HID had a population of 138,418 residents, of whom 10.7% were foreign-born, and the unemployment rate was 6.22%. Meanwhile, the LID had a population of 145,523 residents, of which 18.0% were foreign-born, and the unemployment rate was 13.56%. 

### 2.2. Study Design

**Figure 1:**
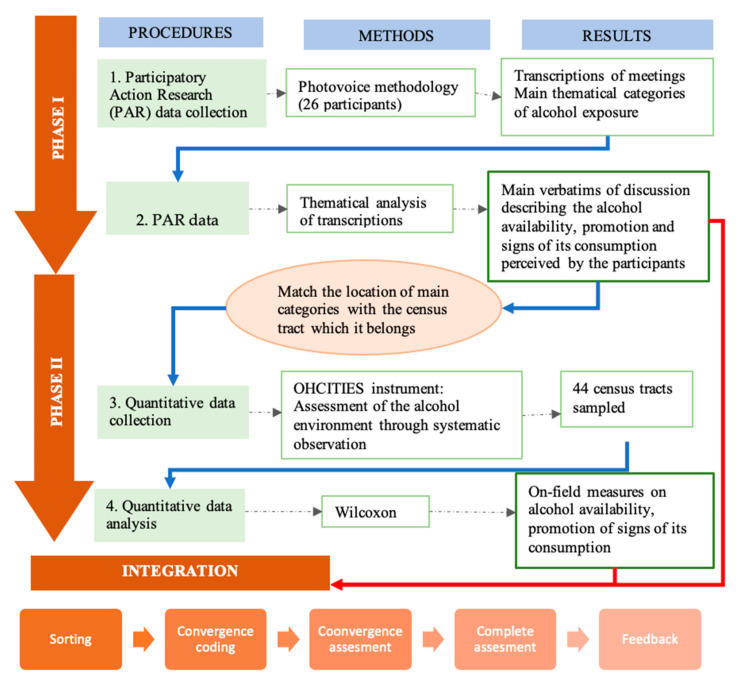
Flow diagram describing the mixed methods approach.

This study was organized in two phases according to a sequential exploratory design [41,46]. First, we used Photovoice to measure residents’ perceived alcohol environment [40]. Second, according to the Photovoice findings, we used systematic social observation to obtain on-field measures on the alcohol availability, its promotion, and signs of its consumption [31]. Figure 1 shows the design of the study. 

### 2.3. Phase I: Photovoice Methodology

Photovoice uses photographs taken by participants with the aim of exploring a community issue [35]. The photographs are discussed providing critical dialogues around participants’ perspectives [47]. In this study, participants photographed and discussed the alcohol environment in their districts [47,48]. 

#### 2.3.1. Participants

The inclusion criteria were adults who (I) had lived in the district for more than one year; (II) spoke Spanish; (III) agreed to attend, at least, five discussion sessions; and (IV) were aged between 40 and 75 years old. These ages were selected because this study is ancillary in the ‘Heart Healthy Hoods’ (HHH) project, aiming to understand how the urban environment may affect cardiovascular health in adult populations [49]. 

Recruitment was led by the research team and the Public Health Technicians. In the end, we had four different groups of participants: two groups by district separated according to sex.

We recruited 7 women and 6 men per district. The median age of participants in the HID was 59 years (ranged 50 to 68). Most of them (n = 12) had a high educational level (bachelor to postgraduate) and had a monthly household income between 2200€ and 2700€ (n = 5). In the LID, the median age was 57 years (ranged 53 to 59). Most of them (n = 5) had a high school diploma (n = 8) and had a monthly household income lower than 1200€ (n = 6). 

#### 2.3.2. Structure of the Photovoice Meetings

Sessions in the HID took place between June and July 2017, and in the LID, they took place between May and June 2018. These sessions were developed in order to conserve the seasonal variation of the alcohol urban environment. There were no alcohol policy changes during this time. The meetings took place weekly and lasted approximately 2 h. Two researchers facilitated the sessions to ensure an equitable engagement of all participants [34,35,47,50]. 

During the first meeting, the purpose of the project was outlined, followed by a photography workshop. We collected informed consent, image release forms, and a brief socio-demographic questionnaire [34,51]. Participants were asked every week to take photos of alcohol in their districts.

During the next sessions, participants discussed the contents of the photographs. All of the sessions were recorded and transcribed for analysis.

#### 2.3.3. Qualitative Analysis

We used a qualitative descriptive [52] and thematic analysis approach [53] to analyze the transcriptions. Three researchers read and coded all the transcripts using predefined codes based on the general theoretical background on alcohol environment framework. This framework comprised the relationship between alcohol consumption patterns and alcohol urban characteristics including alcohol availability, alcohol promotion, and signs of alcohol consumption [7]. 

### 2.4. Phase II: Social Systematic Observation

#### 2.4.1. Setting and Sample Size

Participants provided the addresses of their captured images. We geolocated the images and extracted the census tracts where these photos were located. We included 44 census tracts (25 in HID and 19 in LID) to conduct social systematic observation in order to characterize objectively participants’ perceptions on the urban alcohol environment. 

#### 2.4.2. OHCITIES Instrument

We used the OHCITIES instrument to record on-field data through social systematic observation [31]. This instrument had already been used in previous studies [5,32]. OHCITIES has been proved to be a reliable instrument to capture alcohol availability, its promotion, and signs of its consumption [31].

Two observers were trained for the fieldwork. They completed the OHCITIES instrument walking along all sides of the chosen census tract. Data collection in the HID was conducted between April and May 2018, and in the LID, it was conducted between September and October 2018. The dates in where data were collected were selected in order to minimize coordination times between the methodologies. Fieldwork was conducted on weekdays between 4 and 9 pm.

#### 2.4.3. Alcohol-Related Variables

We collected information on every alcohol outlet within the selected census tract. We also registered alcohol promotion and signs of its consumption in public spaces, beyond the alcohol outlets.

The instrument discriminates among different alcohol outlets. On-premise outlets were defined as places where the alcohol could be consumed (i.e., bars or restaurants). Off-premise outlets were defined as places where the alcohol could be purchased for consumption off the premises (i.e., supermarkets or convenience stores).

We captured the outlet opening hours from signs at their entrances. Where this information was not provided, we attribute the mode of the hours of sale of the outlets within the same census tract and the same type of outlet. Based on the opening hours regulation of the city of Madrid [54], we derived three variables: (1) outlets open (yes/no) during the morning (6 a.m. to 2 p.m.); (2) outlets open (yes/no) during the evening (3 p.m. to 10 p.m.), and (3) outlets open (yes/no) during the night (11 p.m. to 5 a.m.). We defined the availability variables as the number of alcohol outlets open at morning, evening, or night per 1000 inhabitants of the census tract.

Variables related to promotion of alcohol associated with alcohol outlets included the presence (yes/no) of structural elements (i.e., branded awnings with alcohol, label and/or specific alcohol beverages menu), alcohol products (bottles and cans of alcohol or alcohol taps) visible from outdoors (inside the venue and/or in shop windows), and advertisement or sponsorship visible in windows. For on-premise alcohol outlets, we also collected the presence of furniture branded with alcohol in the terraces or outdoor areas of the outlets (i.e., tables, chairs, umbrellas, napkin holders, or ashtrays). We derived a variable summarizing the alcohol elements described above as the presence of at least one sign of alcohol promotion. Promotion variables related to alcohol outlets were expressed as the number of alcohol outlets with the presence of alcohol exposure elements per 1000 inhabitants of the census tract.

The data on the population within each census tract were extracted from the Madrid Municipal Registry in January 2018.

We recorded signs of alcohol consumption as the presence (yes/no) of bottles and/or cans, people drinking alcohol, or other signs of consumption (disposable cups) on the streets, beyond the outlets. We composited a new variable that summarize the presence (yes/no) of at least one sign of alcohol consumption. We expressed the densities of signs of alcohol consumption on the streets as the number of signs of consumption identified along one kilometer of roadway within the census tract sampled, including both sidewalks. The kilometers sampled per census tract were calculated using a network of streets on ArcGIS v.10.6 software (ESRI, Redlands, CA, USA).

#### 2.4.4. Quantitative Analysis

We performed descriptive analysis for alcohol-related variables and compared its distribution according to the districts using the Wilcoxon test with a confidence level of 95%. The analyses were conducted using STATA v.12.0 software (StataCorp LLC, College Station, TX, USA).

### 2.5. Integration of Photovoice and OHCITIES Results

We integrated qualitative and quantitative results using a data triangulation approach. This methodology aims to validate the results from different approaches [55] giving equal priority to both sources of data [38,56].

We used a modified version of the triangulation protocol [55] that had been already used to integrate qualitative and quantitative data [57]. This version is based on five steps: (1) sorting; (2) convergence coding; (3) convergence assessment; (4) complete assessment; and (5) feedback.

#### 2.5.1. Sorting

In this step, findings from Photovoice and social systematic observation were analyzed using the theoretical categories through the alcohol environmental framework used at qualitative analysis and the development of the OHCITIES instrument (alcohol availability, promotion, and signs of alcohol consumption) [7,31].

#### 2.5.2. Convergence Coding

We explored the level of agreement between approaches. The results had “agreement” when alcohol characteristics described by the participants matched with the results found on-field; “partial agreement” when alcohol features captured by one of the methodologies were partially captured by the other one; “silence” when alcohol characteristics were observed by one of the methodologies but not captured by the other one; or “dissonance” when the results obtained for the same alcohol characteristic did not match between methodologies.

#### 2.5.3. Convergence Assessment

We assessed the level of converging findings between methodologies (according to the results obtained that were in agreement or partially in agreement) or dissonant findings (according to the results obtained that were silent or dissonant).

#### 2.5.4. Complete Assessment 

We compared the nature of each methodological scope to enhance the study of key differences in relation to alcohol environment (e.g., since the instrument did not measured noise, we could not evaluate the relationship between its presence and alcohol exposure among the districts. However, it did not mean that this relation did not exist).

#### 2.5.5. Feedback

We shared the triangulated results with the research team to discuss other possible interpretations of the data.

## 3. Results

In total, 281 photographs were analyzed by the participants (139 taken in the HID and 142 in the LID).

We registered a total of 452 alcohol outlets in on-field visits (319 in the HID and 133 in the LID) within the 44 census tracts sampled (25 in the HID and 19 in the LID). Table 1 shows the elements collected during the fieldwork.

### 3.1. Alcohol Availability

In both districts, residents described a high presence of alcohol outlets and wide opening hours. Participants in the HID identified multiple types of alcohol outlets adapted to citizen’s needs (i.e., bars with alcohol discounts addressed to young people or university students or “premium alcohol outlets” addressed to residents with higher socioeconomic status). They also emphasized the high presence of on-premise alcohol outlets in their district targeted to leisure and tourists.

**Figure 2:**
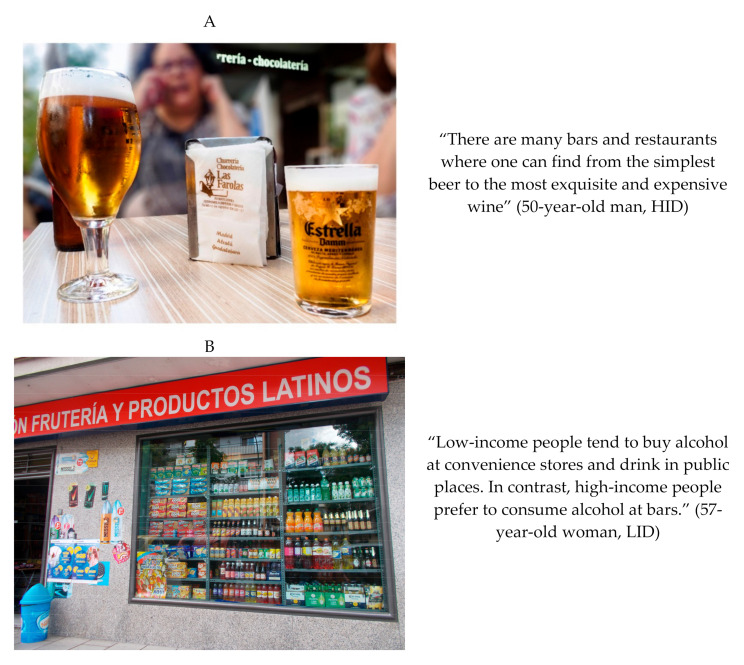
(A) Perceived alcohol availability in a high-income level neighborhood. Photograph: “If you don’t sell alcohol, you don’t sell”. (**B**) Perceived alcohol availability in low-income level neighborhood. Photograph: “A tolerated vice”. Examples of the availability results obtained in the high-income district (HID, Chamberí) (**A**) and in the low-income district (LID, Villaverde) (**B**) through Photovoice.

In the LID, participants reported that the alcohol environment in the district had changed in the last years. Due to the economic crisis, the number of off-premise outlets increased, especially convenience stores. These places offer alcohol beverages cheaper than in bars. However, participants perceived bars as part of their social life (Figure 2).

**Figure 3:**
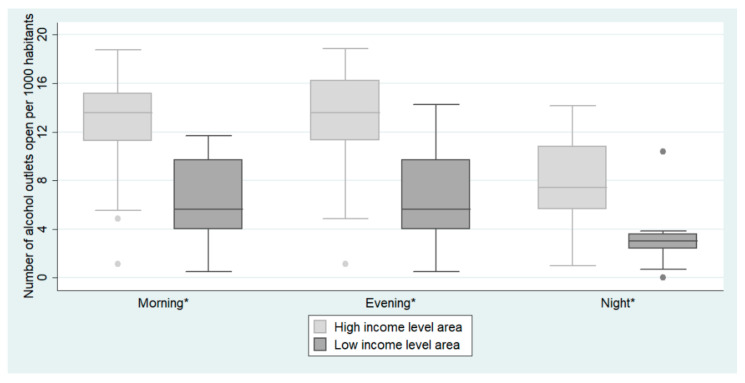
Distribution of the availability of alcohol according to the income level of the district in the city of Madrid, Spain, in 2018. Availability was measured as the number of outlets that were open: (1) In the morning (from 6 a.m. to 2 p.m.); (2) In the evening (from 3 p.m. to 10 p.m.); (3) At night (from 11 p.m. to 5 a.m.); per 1000 inhabitants. * Significant differences. *p*-values were estimated with a Wilcoxon test between the high-income level area (leisure area) and low-income level area (residential area) with a level of confidence of 95%.

We found wider opening hours in the HID than in the LID (all *p*-values < 0.001) (Figure 3). On-premise alcohol outlets were more prevalent than off-premise alcohol outlets in both districts (Table 1). When we compared the proportion of bars and convenience stores between the two districts, we found a higher proportion of bars in the HID than in the LID (52.04% vs. 36.09%, respectively). The proportion of convenience stores was higher in the LID than in the HID (26.32% vs. 11.29%, respectively). 

We found great convergence between the different approaches of alcohol availability in both districts. Perceived and objective measures showed a high availability of alcohol outlets in both districts. 

### 3.2. Alcohol Promotion

At the beginning, most of the participants in the HID did not report alcohol promotion in their districts. As the project progressed, their awareness about the presence of alcohol promotion increased. In the LID, participants discussed alcohol promotion from the beginning, and this issue was more recurrent throughout the sessions. 

**Figure 4:**
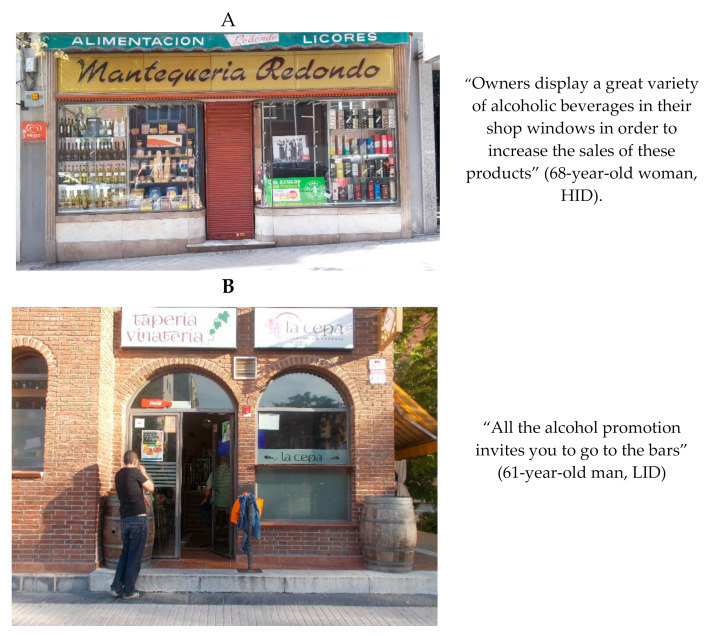
(**A**) Alcohol promotion perceived in high income level neighborhood. Photograph: “Alcohol at first sight”. (**B**) Alcohol promotion perceived in low income level neighborhood. Photograph: “Let´s have the last one”. Examples of the promotion results obtained in the high-income district (HID, Chamberí) (**A**) and in the low-income district (LID, Villaverde) (**B**) through Photovoice.

Participants in both districts highlighted the promotional elements in the terraces of on-premise outlets (such as tables, chairs, or umbrellas featuring alcohol promotions) and described it as a facilitator of alcohol consumption. Figure 4 shows examples of the promotion results obtained in HID (Chamberí) and in LID (Villaverde) through Photovoice. 

**Figure 5:**
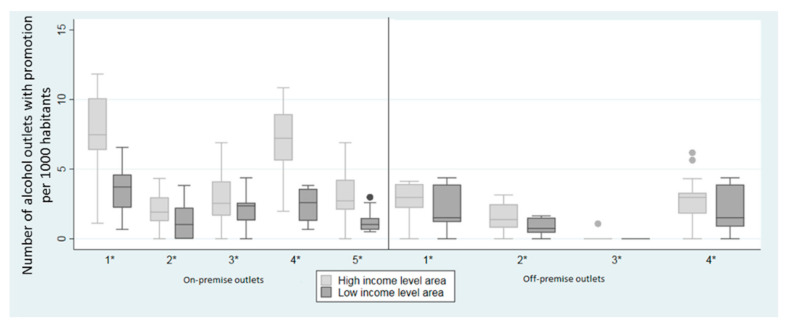
Distribution of the alcohol promotion according to the income level of the district in the city of Madrid, Spain, in 2018. It was measured as: (1) Number of (at least one) signs of alcohol promotion; (2) Number of alcohol outlets with the presence of advertisements; (3) Number of alcohol outlets with the presence of structural elements (awnings, labels, and/or specific alcohol beverage menu); (4) Number of alcohol outlets with alcohol products (alcohol bottles or cans) exhibited in shop windows and/or inside the venue visible from outdoors, and/or (5) alcohol outlets with the presence of furniture (i.e., barrels, alcohol boxes, tables, chairs, umbrellas, napkin holders, or ashtrays) associated with an alcohol brand. Furniture and terraces were recorded only in on-premise alcohol outlets. All the measures were expressed as the number of alcohol outlets with the presence of alcohol promotion element per 1000 inhabitants. * Significant differences. *p*-value were estimated with a Wilcoxon test between high-income level area (leisure area) and low-income level area (residential area) with a level of confidence of 95%.

We found a higher density of promotional elements per 1000 inhabitants (Figure 5) in the HID, independently of the type of promotion (all *p*-values < 0.001). In both districts, the most prevalent elements of alcohol promotion associated to alcohol outlets (Table 1) were the presence of alcohol products inside the venue or in shop windows but visible from outdoors, both in on- and off-premise alcohol outlets. For on-premise alcohol outlets, this was followed by the prevalence of structural elements and furniture in outdoor terraces branded with alcohol. However, participants in both districts reported more frequently the latter. There were dissonances between the perceived and objective measures for alcohol promotion. Whilst alcohol promotion was higher in the HID, residents in the LID were more aware of its presence.

### 3.3. Signs of Alcohol Consumption

**Figure 6:**
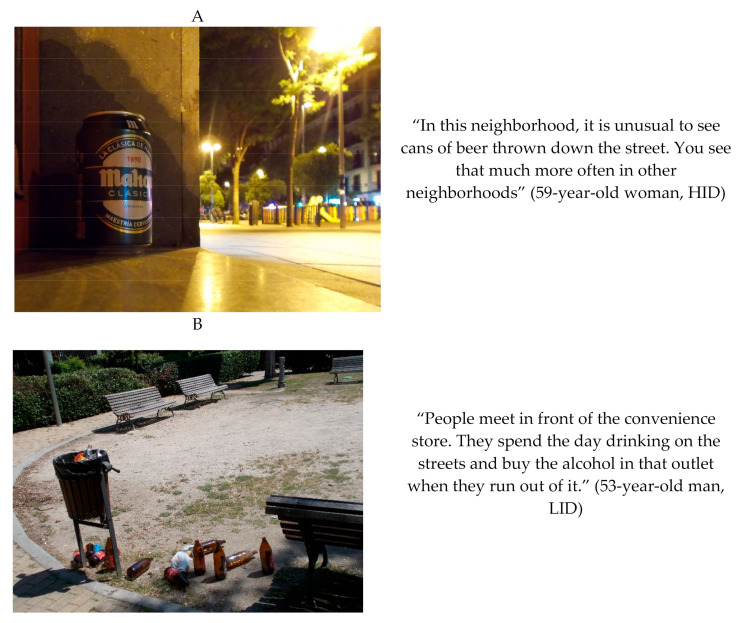
(**A**) Signs of alcohol consumption perceived in high-income level neighborhood. Photograph: “The loner can”. (**B**) Signs of alcohol consumption perceived in low-income level neighborhood. Photograph: “More bins are missing in the streets!” Examples of the signs of alcohol consumption results obtained in the high-income district (HID, Chamberí) (**A**) and in the low-income district (LID, Villaverde) (**B**) through Photovoice.

Participants in the HID perceived the presence of alcohol-related litter as a non-remarkable problem in their district. Nonetheless, some participants claimed that one could still find occasional signs of alcohol consumption on the streets associated with people who use the district for leisure time (Figure 6).

Contrary, most participants in the LID claimed that alcohol consumption in public spaces was common in their district. They described extensively litter related to alcohol as annoying and a driver of antisocial behaviors (Figure 6). Participants argued that this phenomenon is related to the availability of convenience stores and cheaper alternatives to consume alcohol on streets. Moreover, they considered that alcohol consumption in the public spaces could be explained by the income level of the district. This issue may distinguish low-income districts from high-income ones.

**Figure 7:**
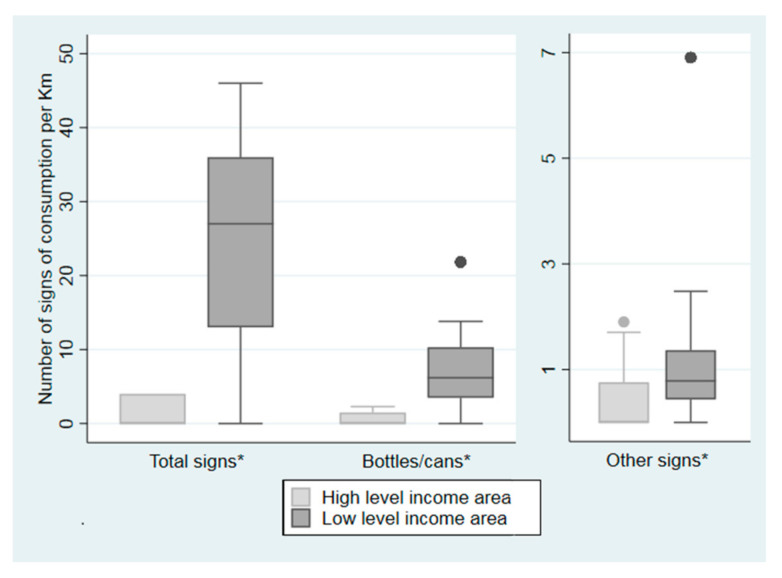
Distribution of the signs of alcohol consumption according to the income level of the district in the city of Madrid, Spain, in 2018. It was measured as: (1) Total signs of alcohol consumption (bottles and/or cans, people drinking alcohol or disposable cups) identified along one kilometer of roadway within the census tract; (2) Bottles and/or cans identified in along one kilometer of roadway within the census tract; (3) Other signs of alcohol consumption different from bottles or cans such as people drinking alcohol or disposable cups identified along one kilometer of roadway within the census tract. * Significant differences. *p*-value were estimated with a Wilcoxon test between a high-income level area (leisure area) and a low-income level area (residential area) with a level of confidence of 95%.

We found 309 signs of alcohol consumption on-field (15 in the HID and 294 in the LID). In both districts, the most prevalent sign of alcohol consumption was the presence of bottles and/or cans (73.33% in HID and 91.50% in LID). When we explored the signs of alcohol consumption per kilometer of roadway (Figure 7), we found that at all of the elements studied were higher in the LID (*p* = 0.011 for people drinking on the street and *p* < 0.001 for the rest of the signs of consumption). 

Most results showed convergence. On-field data of litter related to alcohol and alcohol consumption in the street agreed with the reported exposure to these signs of alcohol consumption on streets in both districts. However, the antisocial behaviors and the annoying noise of alcohol consumption on the street described by participants were silent on-field measures, since the OHCITIES instrument did not include this information. 

## 4. Discussion

We used a mixed methods approach to characterize the alcohol environment of different income-level districts in Madrid. We also compared the differences and similarities between residents’ perceptions and objective measures. 

Perceived and on-field observations found a high availability of alcohol in both districts. This kind of perception contributes to the belief that alcohol consumption is socially accepted [58]. In addition, previous studies had found a positive association between a high perception of availability and hazardous drinking patterns [10,59]. HID participants identified different types of alcohol outlets according to the user. In agreement with the theory of Bourdieu, people with more cultural capital (wealthy or with a high educational level) promote a hierarchical distinction of the consumers according with their tastes, as an indicator of social class [60]. 

In both districts, bars were the most prevalent type of alcohol outlet. There were well described by all the participants as essential meeting points with their relatives and friends. Bars have been shown to provide more opportunities to drink and socialize outside home [20]. In the LID, participants also highlighted the role of off-premise in their district as a place where alcohol products have more affordable prices than in the bar. Thus, off-premise alcohol outlets, and particularly convenience stores, were more prevalent in the LID than in the HID. An unequal distribution of alcohol outlet typologies according to income-level may promote different effects on alcohol behaviors.

Alcohol promotion was undetected by some Photovoice groups in comparison to the results obtained from the objective measures. Whilst alcohol promotion was higher in the HID, residents in the LID discussed promotion more deeply.

Participants in both districts highlighted alcohol promotion targeted to young people. Indeed, the alcohol industry adjusts their message according to the age, ethnicity, or income of the audience [61,62,63,64]. The advertisements targeted to the adult population were underreported by the participants, especially in the HID, where the presence of this type of promotion was higher than in the LID. This misperception could be the result of the repeated alcohol messages promoted by the alcohol industry. According to SCT, when the same information is processed repeatedly and consciously, the information is integrated through an “automatic cognitive process” [12,13]. Thus, the quantity of alcohol promotion we objectively measured on-field was much higher than what they reported. 

The European Union has statutory acts to control the exposure to alcohol promotion [65]. However, the alcohol industry has found strategies to promote their beverages. Future interventions may enforce and strengthen the laws to protect the population from alcohol promotion exposure given its association with higher alcohol consumption [24,62,66,67,68,69].

Residents in the LID reported more frequently the presence of signs of alcohol consumption than in the HID. These results agreed with on-field measures. The presence of litter in deprived areas has been related with limited resources devoted to cleaning the streets [70,71]. Moreover, participants described these signs of alcohol as drivers of antisocial behaviours and bad use of public spaces. Similar results had been found in previous research [72,73,74]. Thus, its presence had been used in other studies as an indicator of the devaluation of an area [73,75].

In addition, the presence of people drinking on the streets was higher in the LID than in the HID. Participants in this district identified that the higher presence of off-premise outlets might facilitate alcohol consumption in public spaces. Previous studies supported this idea [61,73,76]. The high presence of people drinking in the streets could influence the perception that some participants from the LID consider outdoor public spaces as the place where they socialize [7,20]. Some countries, such as Spain (Law 5/2018) or the United Kingdom (Anti-social Behavior, Crime and Policing Act 2014), have banned alcohol consumption in public spaces. However, our results suggested that this regulation is not enforced, especially in the LID. The unequal distribution of signs of alcohol consumption may contribute to generate health inequalities related with alcohol consumption.

Our study presents some limitations. First, the photographs could be taken anytime, while social systematic observation was conducted between 4 p.m. and 9 p.m. We choose this time frame to ensure that most of the outlets were opened. Second, aspects related to alcohol affordability (i.e., prices of alcohol or alcohol illegal sales), or the annoyance caused by noises of people drinking on the streets are not included in the present study. Those aspects appeared in some Photovoice group discussions but were not included in the OHCITIES instrument. Thus, these topics are not compared in this study. Finally, our purposive sampling design in the Photovoice may limit generalizability. It would be interesting in future studies to include participants from other nationalities and ages.

## 5. Conclusions

This study has helped improve our understanding of the alcohol environment. The use of mixed methods allows us to explain the role of alcohol in the daily lives of participants and show results impossible to observe with the use of just one methodology. 

We found similarities and discrepancies between the residents’ perceptions and the on-field objectives measures in terms of availability, promotion, and signs of consumption. Moreover, we found differences according to area income level. This approach may help us design more effective public health interventions in order to decrease harmful drinking and health inequalities. 

## Figures and Tables

**Table 1:** Distribution of alcohol features associated to alcohol outlets objectively measured within 44 census tracts in two different income-level districts in the city of Madrid, Spain, in 2018.

	Total		High Income-Level		Low Income Level	
	N	%	N	%	N	%
Number of census section selected	44	-	25	-	19	-
ALCOHOL AVAILABILITY						
Total alcohol outlets	452	100.00	319	100.00	133	100.00
On-premise	322	71.24	236	73.98	86	64.66
Bar	214	47.34	166	52.04	48	36.09
Restaurant	86	19.03	63	19.74	23	17.29
Night clubs	10	2.21	5	1.57	5	3.76
Others	12	2.66	2	0.63	10	7.52
Off-premise	130	28.76	83	26.02	47	35.34
Supermarkets	27	5.97	17	5.33	10	7.52
Convenience stores	71	15.71	36	11.29	35	26.32
Specialty stores	29	6.42	27	8.46	2	1.50
Wine or liquor stores	2	0.44	2	0.63	-	-
Others	1	0.22	1	0.31	-	-
ALCOHOL PROMOTION						
On-premise outlets						
With presence	252	55.75	183	57.37	69	51.88
Advertisements and sponsorship in shop window or visible windows						
Present	69	15.27	45	14.10	24	18.04
Structural elements associated with alcohol products						
Present	116	25.66	73	22.88	43	32.33
Furniture elements associated with alcohol products						
Present	98	21.68	70	21.94	28	21.05
Bottles and/or cans inside the venue or in shop windows visible from outdoors						
Present	218	48.23	169	52.98	49	36.84
Off-premise outlets						
With presence	100	22.12	62	19.44	38	28.57
Advertisements and sponsorship in shop window or visible windows						
Present	48	10.62	30	9.40	18	13.53
Structural elements associated with alcohol products						
Present	1	0,22	1	0.31	-	-
Bottles and/or cans inside the venue or in shop windows visible from outdoors						
Present	100	22.13	66	20.69	34	25.56
